# Multiple Bone Metastases as the First Manifestation of Hepatocellular Carcinoma in Patient with Noncirrhotic Liver

**DOI:** 10.1155/2015/512849

**Published:** 2015-11-09

**Authors:** Soo Ya Bae, Hyun Jung Kim, Hyun Ho Oh, Min Kwan Kwon, Jong Ho Lee, Moon Park, Byeong Seok Sohn

**Affiliations:** ^1^Department of Internal Medicine, Sanggye Paik Hospital, Inje University College of Medicine, Seoul 139-707, Republic of Korea; ^2^Department of Pathology, Sanggye Paik Hospital, Inje University College of Medicine, Seoul 139-707, Republic of Korea

## Abstract

Hepatocellular carcinoma (HCC) generally occurs on the background of chronic liver disease. Chronic hepatitides B and C and alcoholic liver disease are well-known risk factors for HCC, and it is uncommon in noncirrhotic liver. Extrahepatic metastasis seldom occurs in patients with early stage intrahepatic HCC and isolated bone metastases as a first documented extrahepatic metastasis is unusual presentation. In this report, we present a rare case of small solitary HCC (<3 cm) in noncirrhotic liver, presenting isolated bone metastases as a sole manifestation in patient with no well-known risk factors. This case suggests that HCC should be considered as one of differential diagnoses in patient presenting with multiple bone metastases, even in the absence of liver cirrhosis.

## 1. Introduction 

Up to 10% of patients with cancer could develop symptomatic secondary spinal metastasis [1, 2]. Musculoskeletal system is the 3rd most common metastatic site following lung and liver. Cancers from breast, lung, prostate, hematopoietic (lymphoma or multiple myeloma), and renal origins account for the vast majority of extradural spinal metastases [1, 2]. Breast, prostate, thyroid, lung, and kidney are the most commonly found primary cancer, and meticulous history taking, physical examination, laboratory examinations, and imaging studies are needed to reveal primary tumor [3].

Hepatocellular carcinoma (HCC) is the 5th most common primary malignancy and it has 2nd highest mortality rate in South Korea [4]. Chronic hepatitis B viral infection, followed by alcoholic liver disease and chronic hepatitis C viral infection, is the common cause of HCC and well-known risk factors of it. About 5% of HCC is known to be cryptogenic, and nonalcoholic fatty liver disease (NAFLD) has been considered as a representative cause of cryptogenic HCC in South Korea [5]. Since extrahepatic metastases of HCC are relatively rare at the time of initial diagnosis, diagnosis procedures for extrahepatic metastases have not been standardized [6]. The most frequent site of extrahepatic metastasis is lung, followed by lymph node, bone, and adrenal gland [7]. The most common location of bone metastasis is spine, followed by pelvis and rib [6, 8, 9]. In spine, the most frequently involved site of metastases is lumbosacral vertebrae, followed by thoracic vertebrae and cervical vertebrae [10, 11].

In this report, we report a rare case of small solitary HCC (<3 cm), presenting isolated bone involvement as a first manifestation of extrahepatic metastasis in patient with noncirrhotic liver and no well-known risk factors.

## 2. Case Presentation

An 83-year-old woman visits our hospital with complaint of a one-week history of worsening lower back pain. She felt lower back pain 3 months ago, occasionally radiating along right leg. She had taken medication for hypertension, diabetes mellitus, and dyslipidemia for several years. She underwent abdominal ultrasonography in our hospital 8 and 3 years ago, and she was suspected to have mild and moderate fatty liver, respectively. She denied alcohol drinking and cigarettes smoking for her entire life. Her family history was uneventful. She admitted for further evaluation.

On admission, her blood pressure was 140/90 mmHg. Height and body weight were 149 cm and 47.3 kg, respectively. Body mass index was 25.8 kg/m^2^. In physical examination, there was direct tenderness on sternum, right ribs, and thoracic and lumbar spine. Hypoesthesia on L4/5 dermatome was noted. At left occiput, a 3 cm-sized, fixed, and round mass was palpated without tenderness. She said that it seemed to be felt about 3 months ago. Breath sound was clear without adventitious sound, and heart beat was regular without murmur. There were no abnormal findings in abdomen. There was no palpable lymph node. The remaining examinations are unremarkable.

In laboratory examinations, total leukocyte count was 7,440/mm^3^, the level of hemoglobin was 11.3 g/dL, and platelet count was 245,000/mm^3^. In chemistry battery, creatinine was 1.2 mg/dL, uric acid was 5.5 mg/dL, total protein was 7.0 g/dL, albumin was 3.9 g/dL, AST was 38 IU/L, AST was 26 IU/L, total bilirubin was 0.2 mg/dL, alkaline phosphatase was 135 IU/L, and serum total calcium was 9.0 mg/dL. In lipid battery, total cholesterol was 130 mg/dL, triglyceride was 75 mg/dL, HDL cholesterol was 40 mg/dL, LDL cholesterol was 82 mg/dL, and HbA1c was 6.2%. In serologic test, hepatitis B surface antigen, hepatitis B core antibody, and hepatitis C antibody were negative; and hepatitis B surface antibody was positive. Serology for HIV and syphilis were negative.

**Figure 1:**
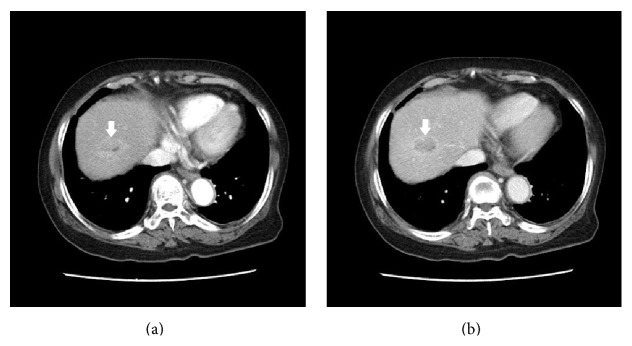
(a) Arterial phase, faintly enhanced mass. (b) Portal vein phase, hypodense nodule.

**Figure 2:**
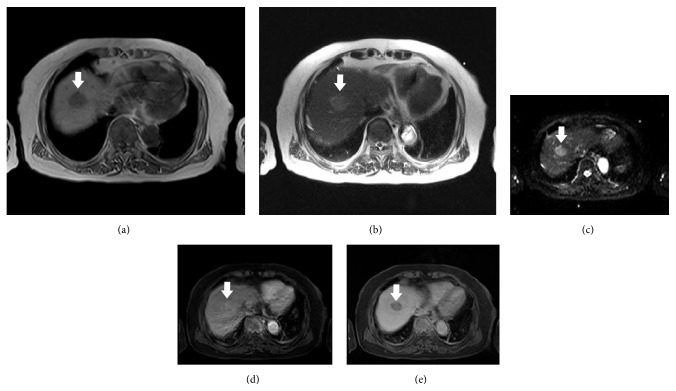
(a) Low signal intensity on T1. (b) High signal intensity on T2. (c) Diffusion restriction positive. (d) Arterial enhancement. (e) Delayed washout (5 min).

In whole spine magnetic resonance image (MRI), 3 cm-sized metastatic lesions were revealed in T12 and L5 body to right pedicle, respectively. Also, small nodular lesions at T3, 9, 10, and 3 cm-sized occipital lesion were observed. To find primary cancer, chest computed tomography (chest CT) and abdominopelvic CT (AP-CT) were performed. In chest CT, multiple bone metastases were shown in sternum, right clavicle, right 11th rib, and thoracic spines. However, there was no abnormal finding to suspect primary cancer. In AP-CT, small round nodule was observed in liver segment 8, which was measured by 2.5 cm, showing faint arterial enhancement and delayed washout (Figure 1). Also, moderate degree of fatty liver was suspected concordantly with her previous findings in ultrasonography. However, there were no features of cirrhosis such as surface nodularity, ascites, or splenomegaly. Also, there was right ovarian cystic tumor without mural nodules or calcification. It was measured by 3 cm compared to 1.4 cm 8 years ago. Liver MRI and *α*-fetoprotein (AFP) were checked due to the typical enhancing pattern observed in AP-CT. Carbohydrate antigen 125 (CA-125) also was checked due to right ovarian cystic tumor despite the fact that it did not show hot-uptake in PET-CT. In liver MRI, hepatic mass (<3 cm) showed low signal intensity on T1WI, high signal intensity on T2WI, diffusion restriction positive, arterial enhancement, delay washout, and defect on hepatobiliary phase in segment 8 (Figure 2). The level of AFP was elevated to 12230 ng/mL, and the level of CA-125 was in normal range. HCC was identified; however, it was a small solitary HCC with T2 intrahepatic stage in modified International Union Against Cancer (UICC) staging system. There was no other intrahepatic metastasis. Because the liver lesion or ovarian lesion was too small to conclude it as the primary cancer of the disseminated bone metastases, further evaluation with positron emission tomography-computed tomography (PET-CT) was planned. PET-CT image showed fluorodeoxyglucose (^18^F-FDG) hot-uptakes in hepatic segment 8 (max standardized uptake value: 3.7), multiple bone metastases at right scapula, sternum, cervix—thoracic—lumbar vertebrae, bilateral ribs, left occipital bone, and right mandible. However, no other abnormal ^18^F-FDG uptakes were shown except liver and bone lesions. No lung metastasis and lymph node metastasis were suspected.

**Figure 3:**
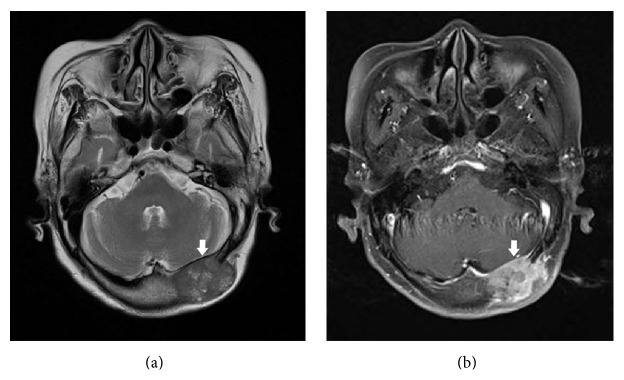
(a) T2W and FLAIR axial image. (b) T1W axial image with Gd-DTPA. Relatively well-defined lobulating, enhancing skull mass in left occiput (3.5 × 2.5 cm), with destruction of bone and intracranial extension compressing adjacent sinus, but no definite brain invasion.

**Figure 4:**
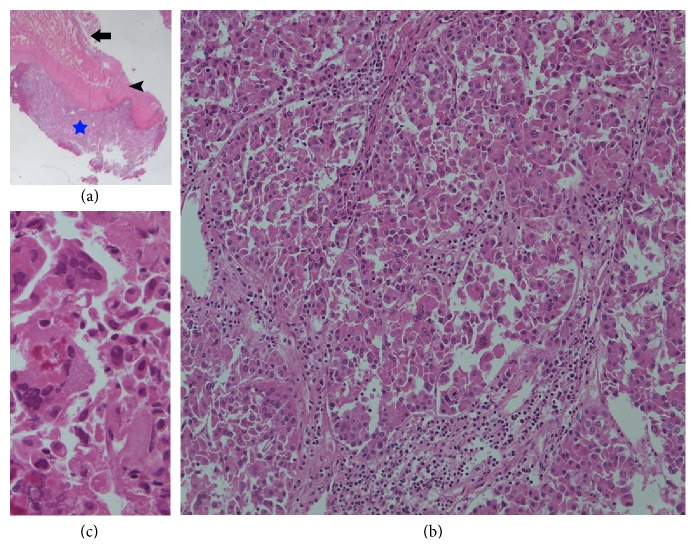
(a) A nodular proliferating mass (blue star) under the skeletal muscle (thick arrow) and fascia (arrow head) (H&E, ×10). (b) The tumor reveals trabecular or acinar arrangement surrounded by sinusoidal vessels (H&E, ×200). The individual tumor cells are hepatoid (thick eosinophilic and granular cytoplasm, round central nuclei, prominent nucleoli). (c) Some tumor cells are pleomorphic with frequent multinucleation (Edmondson-Steiner Nuclear grade 4/4) (H&E, ×400).

Because it is very unusual clinical finding that the diffuse and extensive musculoskeletal metastases were derived from small solitary HCC or ovarian cystic mass, we planned tissue biopsy at bone metastatic site to confirm the primary cancer. Brain MRI was taken to biopsy at occipital mass; brain MRI showed lobulating, enhancing skull mass in left occiput (3.5 × 2.5 cm), with destruction of bone and intracranial extension (Figure 3). Incisional biopsy from occiput mass was done, and it revealed metastatic HCC with Edmondson Steiner grade 4/4 (Figure 4). Finally, HCC with multiple bone metastases, modified UICC T2N0M1, IV-B stage was diagnosed. The patient needs two times of resuture due to the wound dehiscence at the incision site.

After diagnosis, the cause of HCC was searched again. However, antinuclear antibody, anti-neutrophil cytoplasmic antibody, anti-liver kidney microsomal antibody, anti-mitochondrial antibody, anti-smooth muscle antibody were all negative findings. Immunoglobulin G was in normal range (1461 mg/dL), serum ceruloplasmin was 32.7 mg/dL, and ferritin was 79.24 ng/mL. Although there was a suspicion of fatty liver and associated NAFLD, we did not perform liver biopsy because the patient would not have additional benefit from it.

With regard to the advanced stage and patient's poor performance status, conservative treatment with radiation therapy on L5 (39 Gy in 13 fractions) was done. After 1 month from the completion of radiotherapy, she complained of right chest pain and increased occipital mass, additional radiotherapy on metastatic lesion of left occiput and right 11th rib was done. After 3 months from then, PET-CT was reevaluated due to the aggravating lower back pain. Although there was little change in primary cancer in hepatic segment 8, the previous bone metastases were aggravated significantly and multiple new bone metastatic lesions were developed. She wanted supportive care and was transferred to hospice.

## 3. Discussion

Although almost all kinds of cancers can spread to bone, most frequently encountered primary cancers that metastasize to bone are prostate, breast, kidney, lung, and thyroid. When the primary site is ambiguous, the lung and the kidney should be suspected as the site of primary origin [3]. In this case, we did not perform the liver biopsy for the evaluation of primary site. However, there was little difficulty in the diagnosis of the primary cancer which was confirmed by typical findings at liver dynamic CT and MR liver and the elevated AFP level. Microscopic examination of the biopsy specimen from the occiput lesion revealed (1) nodular proliferating mass showing trabecular or acinar arrangement of tumor cells surrounded by sinusoidal vessels, (2) hepatoid cell with thick eosinophilic and granular cytoplasm with prominent nucleoli, and (3) frequent multinucleation in tumor cells. All of these findings are consistent with metastatic HCC rather than hepatoid adenocarcinoma or HCC arising from ectopic liver tissue.

In contrast to intrahepatic metastasis of HCC, extrahepatic metastasis of HCC has been known to be uncommon, and bone metastasis as the first manifestation of extrahepatic HCC seldom occur; therefore only several case reports have been reported. In one study including 149 patients with extrahepatic metastasis of HCC [7], the most frequently involved sites of extrahepatic metastasis were lung, lymph node, musculoskeletal, and adrenal gland in order of frequency. The most of musculoskeletal involvements (66%) already had multiple other nonosseous sites of metastatic disease at the time of manifestation of the first documented extrahepatic HCC. However, the isolated bone metastasis as the first manifestation was only seen in 14 out of 149 (9.5%) patients. The frequent involved location was lumbosacral and thoracic spine. Moreover, it is advocated that HCC has distinctive metastatic pathways. While lung is the most common site of extracranial metastases in the nonskull involvement group, bone is the most common site of extracranial metastases in the skull involvement group. Two metastatic pathways were suggested as the hematogenous route via the lungs to the brain parenchyma and the osseous route via Batson's venous plexus to the skull [9].

Since symptoms attributable to HCC are usually absent in early stage, liver ultrasonography and serum AFP are used for the surveillance of HCC in high risk group. While surgery is the curative and most optimal therapy, for patients ineligible for surgery, locoregional and/or systemic therapies can be applied in anticipation of survival benefit. Among locoregional therapies, percutaneous radiofrequency ablation (RFA), percutaneous ethanol injection (PEI), and transarterial embolization (TACE) were widely accepted [12].

Surveillance of HCC in high risk group and locoregional/systemic therapies to patients ineligible for surgery have prolonged the survival of HCC, and the incidence of extrahepatic metastases seems to be increasing accompanied with the prolonged survival of HCC. Positivity for viral markers, larger tumor diameter, multiple tumor nodules, the presence of vascular invasion, and the elevated tumor markers were associated with the development of extrahepatic metastasis [6, 8, 12]. With regard to larger tumor diameter, these extrahepatic metastasis was associated with intrahepatic HCC stage; a majority (87%) of patients with extrahepatic HCC had intrahepatic stage III (10%) and stage IVA (76%) tumors. Only 10% of the patients with extrahepatic metastasis had intrahepatic stage II tumor according to previous report [7]. Most of HCC occurs on the background of chronic liver disease including chronic hepatitis B and hepatitis C viral infection and alcoholic liver disease ultimately followed by cirrhosis. However, NAFLD, the hepatic manifestation of obesity and related metabolic disorders, is now a known-risk factor of cryptogenic cirrhosis and HCC. Also, HCC may complicate noncirrhotic NAFLD with mild or absent fibrosis [13–15].

We present our experience of a rare case of small solitary HCC (<3 cm) in noncirrhotic liver, presenting isolated bone metastases as a sole manifestation in patient with no well-known risk factors. This case suggests that HCC should be considered as one of differential diagnoses in patient presenting with multiple spine metastases, even in the absence of liver cirrhosis.
